# Metabolomics and Proteomics Responses of Largemouth Bass (*Micropterus salmoides*) Muscle under Organic Selenium Temporary Rearing

**DOI:** 10.3390/molecules28145298

**Published:** 2023-07-09

**Authors:** Hailan Li, Wenbo Liu, Tao Liao, Wei Zheng, Jianhui Qiu, Guangquan Xiong, Xiaoyan Zu

**Affiliations:** 1 Key Laboratory of Cold Chain Logistics Technology for Agro-Product, Ministry of Agriculture and Rural Affairs, Institute of Agro-Products Processing and Nuclear Agricultural Technology, Hubei Academy of Agricultural Sciences, Wuhan 430064, China; hl.li@hbaas.com (H.L.); liuwenbo12323@163.com (W.L.); zxqqx123@163.com (T.L.); qiujianhui2022@163.com (J.Q.); 2 School of Chemical and Environmental Engineering, Wuhan Institute of Technology, Wuhan 430205, China; 3 Institute of Agricultural Economics and Technology, Hubei Academy of Agricultural Sciences, Wuhan 430064, China; oriswa@sina.com

**Keywords:** organic selenium, *Micropterus salmoides* muscle, temporary rearing, metabolomics, proteomics

## Abstract

Organic selenium has been widely studied as a nutritional supplement for animal feed. However, there are few studies on the effect of organic selenium on flesh quality. In this study, the effects of organic selenium (yeast selenium (YS), Se 0.002 mg/L) on the metabolism and protein expression in *Micropterus salmoides* muscle under temporary fasting condition (6 weeks) were investigated. The muscle structure was observed through a microscope, and regulatory pathways were analyzed using proteomics and metabolomics methods. Electron microscopy showed that YS made the muscle fibers of *M. salmoides* more closely aligned. Differential analysis identified 523 lipid molecules and 268 proteins. The numbers of upregulated and downregulated proteins were 178 and 90, respectively, including metabolism (46.15%), cytoskeleton (11.24%) and immune oxidative stress (9.47%), etc. Integrated analyses revealed that YS enhanced muscle glycolysis, the tricarboxylic acid cycle and oxidative phosphorylation metabolism. In the YS group, the content of eicosapentaenoic acid was increased, and that of docosahexaenoic acid was decreased. YS slowed down protein degradation by downregulating ubiquitin and ubiquitin ligase expression. These results suggest that organic selenium can improve *M. salmoides* muscle quality through the aforementioned pathways, which provides potential insights into the improvement of the quality of aquatic products, especially fish.

## 1. Introduction

Muscle tissue is the main edible part of fish, and, hence, the structure, protein and fat content of muscle tissue are important indicators to evaluate the quality of fish flesh [1]. Oh et al. showed that temporary fasting could be used as a more economical way to feed certain farmed fish [2]. Currently, a study on grouper fish has indicated that proper fasting can reduce the fat content of fish and that it is an effective means of improving fish flesh quality [3]. Unlike mammals, fish can survive for a long period of time in a fasted state [4]. However, prolonged fasting may lead to the production of significant amounts of reactive oxygen species, which can destroy biological macromolecules such as proteins, lipids and nucleic acids, thus negatively affecting the quality of fish [5]. Our previous study indicated that the treatment of *Micropterus salmoides* with yeast selenium (YS, containing 2000 mg Se per 1 kg of YS) during 8 weeks of temporary rearing under fasting conditions improved the textural properties of the fish, such as elasticity, recovery and gelatinous properties, and it mitigated the decline in its muscle quality [6].

As an antioxidant, selenium (Se) can protect cells and tissues from oxidative stress and can enter selenium proteins, such as glutathione oxidase and thioredoxin reductase, in the form of selenocysteine and selenomethionine in order to play a biological role in regulating cell oxidation and reduction reactions [7]. The bioavailability of Se is not only closely related to the chemical form, but it is also influenced by the metabolic variability in the species. Different studies on salmon and trout have indicated that organic Se (Met-Se, Yeast-Se) has a higher bioavailability and toleration than inorganic Se (selenate, selenite) [8]. Furthermore, Se improves stress capacity in fish; for example, nano-Se improves the antioxidant status in tilapia [9]. In rainbow trout, organic Se enhances resistance to physical stressors [10] and increases the expression of antiviral defense mediators during viral infections [11]. However, Berntssen et al. found that supplementation of organic selenomethionine was safe at 0.45 mg/kg, but higher doses than 16 mg/kg could damage the liver and kidneys of Atlantic salmon [12]. So, ingesting high doses of selenium not only causes fish poisoning but also is detrimental to the health of consumers. The European Food Safety Authority recommends that the maximum limit for total Se in animal feed be set at 0.2 mg Se/kg feed [13]. The World Health Organization recommends an average daily intake of about 55 μg of Se for adults [14].

Metabolomics and proteomics technologies can effectively monitor the response of fish to environmental changes. Yu et al. evaluated the importance of oxidation in improving the muscle quality of crisp grass carp based on proteomics and metabolomics, so the study of omics has great potential in understanding the biological mechanism of improving fish quality [15,16]. In recent years, the aquacultural production of *M. salmoides* has rapidly increased. Therefore, considering *M. Salmoides* as a model organism, this study investigated the effects of YS on the expression of lipid and protein molecules in the muscle tissues of *M. salmoides* during fasting. Metabolomics and proteomics tools were used to investigate the possible regulatory pathways. Hence, the current study aimed to generate basic data that may help in understanding the mechanism behind the nutritional regulation of fish muscle.

## 2. Results

### 2.1. Morphology of Muscle Fibers 

**Figure 1:**
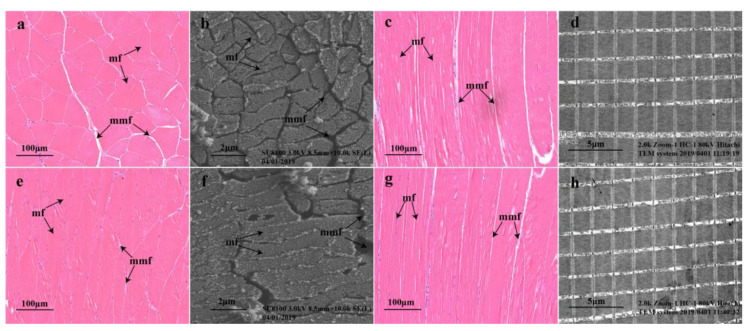
Muscle microstructure of *Micropterus salmoides*. Cross-sectional microstructure of bass in the blank group (**a**,**b**); cross-sectional microstructure of bass in the YS group (**e**,**f**); longitudinal microstructure of bass in the blank group (**c**,**d**) and longitudinal microstructure of bass in the YS group (**g**,**h**). mf, muscle fibers; mmf, matrix between muscle fibers.

The muscle transverse structures, viewed under optical (Figure 1a,e) and scanning electron microscopes (Figure 1b,f) at the same magnification and field of view, suggested that the muscle fibers in the YS group (Figure 1e,f) were more closely arranged than those in the control group. The longitudinal structure of the muscle, indicated by optical (Figure 1c,g) and transmission electron microscopy (Figure 1d,h), suggested that the muscle fiber diameter in the YS group (Figure 1g,h) was smaller than that in the control group.

### 2.2. Metabolomics Response of Muscle Lipids under Se Exposure

**Figure 2:**
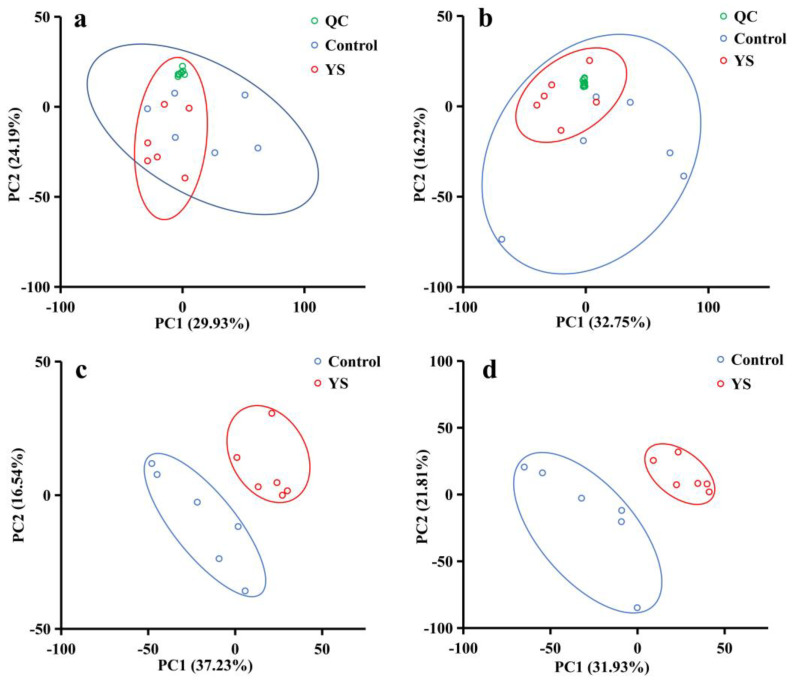
Principal component score plots for quality control (QC) samples (**a**,**b**) and metabolomic characteristics of bass muscle partial least squares discriminant analysis (PLS-DA) score plots (**c**,**d**). (**a**,**b**) present the principal component score plots for ESI^−^ and ESI^+^ QC samples, respectively. (**c**,**d**) are the PLS-DA score plots for ESI^−^ (R^2^ = 0.8758, Q^2^ = 0.5736) and ESI^+^ (R^2^ = 0.9129, Q^2^ = 0.6248), respectively; green represents QC samples, while blue represents control samples (6 weeks of transient incubation), and red represents the YS group (6 weeks of temporary rearing with the addition of yeast Se).

Ions were filtered at a relative standard deviation of ˂30%, and 6772 and 3588 ions were obtained in the ESI^+^ and ESI^−^ modes, respectively. The quality control samples in the principal component analysis were clustered in the center of the scoring plot (Figure 2a,b). The better the aggregation, the more stable the instrument and the better the quality of the data collected, indicating a sound reproducibility of sample extraction and lipid molecular analysis. Figure 2c,d shows that the YS and blank groups were evidently separated and significantly different. According to the model parameters established by PLS-DA, the R^2^ values were 0.9129 and 0.8757 for the ESI^+^ and ESI^−^ modes, respectively, indicating that the current PLS-DA model was reliable. Under the two modes, 697 and 344 difference ions were screened, respectively.

**Figure 3:**
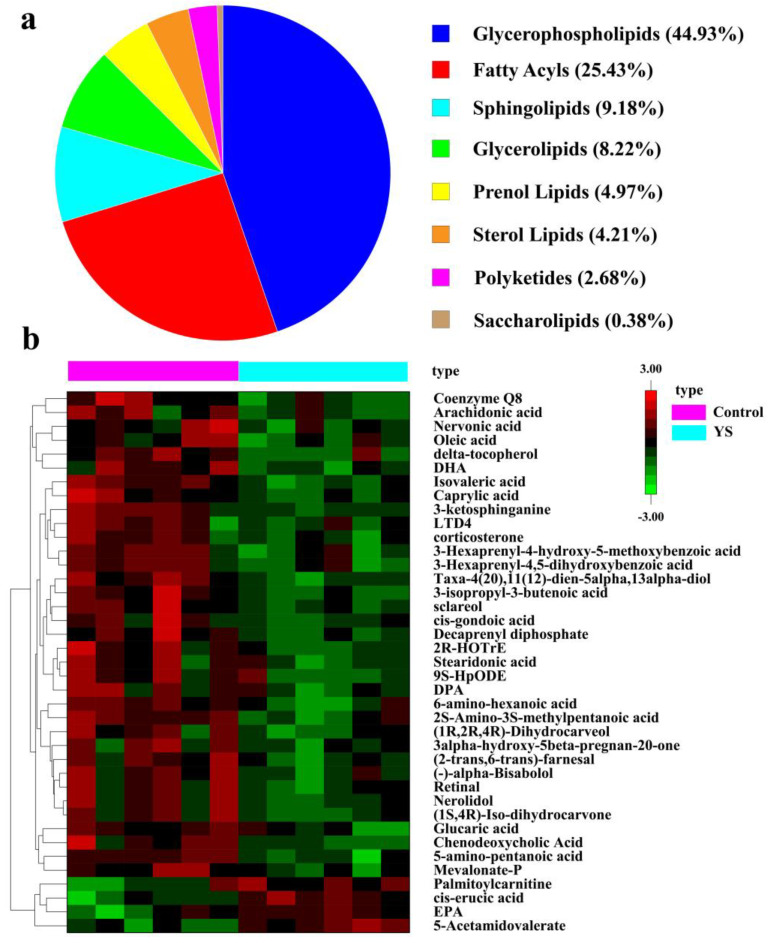
Classification diagram of differential lipid molecules (**a**) and heat map of differential metabolite analysis (**b**). Each column in the plot represents one sample: pink for control group, blue for YS group. Each row represents one lipid molecule. Different colors indicate different expression, with colors from red to green indicating high to low expression, respectively.

The Lipid Maps database was used to annotate the differential ions, and 523 lipid molecules were described. These lipid molecules could be classified into eight classes, namely glycerophospholipids (44.93%), fatty acids (25.43%), sphingolipids (9.18%), glycerolipids (8.22%), prenol lipids (4.97%), sterol lipids (4.21%), polyketides (2.68%) and saccharolipids (0.38%) (Figure 3a). To understand the potential pathways through which YS affects lipid metabolism in *M. salmoides*, we performed KEGG pathway annotations on differential lipid molecules and annotated an aggregate of 17 pathways. The results revealed that YS affected the synthesis pathways of unsaturated fatty acids, in which the levels of eicosapentaenoic acid (EPA) and cis-erucic acid (CEA) increased, while those of docosahexaenoic acid (DHA), docosapentaenoic acid (DPA), nervonic acid (NA), dihomo-γ-linoleic acid (DGLA) and cis-gondoic acid (CGA) decreased. The differences in the expression of the lipid molecules in the annotated pathways in the different groups are presented as a heat map in Figure 3b.

### 2.3. Muscle Proteomic Response under Se Exposure

**Figure 4:**
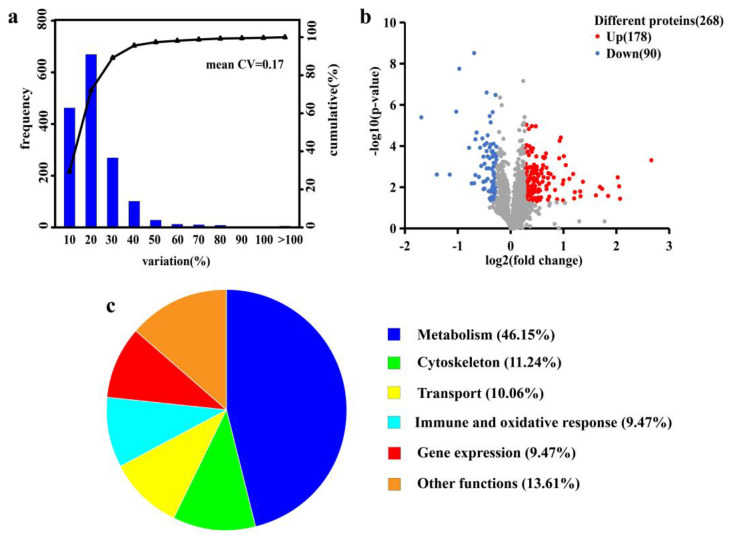
Coefficient of variation (CV) distribution plot of replicate experiments (**a**), volcano plot of significantly differential proteins (**b**) and functional classification of differential proteins (**c**). The X-axis in the plot a denotes the CV distribution range. The Y-axis is the number of proteins in the corresponding CV range: the right-hand side denotes the cumulative proportion of proteins in each CV range. The X-axis in plot b represents the protein difference ploidy taken as log2. The Y-axis denotes the corresponding −log10 (*p*-value). The above satisfies a *p*-value < 0.05 and difference multiples > 1.20 or <0.833, indicating a significantly different protein.

Filtered at a criterion of a 1% false discovery rate, an aggregate of 3669 unique peptides and 1570 proteins were identified in the control and YS groups. The reproducibility of the quantification method was assessed based on the value of the coefficient of variation (CV) (Figure 4a). The CV value was 0.17, indicating a good reproducibility. Based on the *t*-test with a *p*-value < 0.05 and difference multiples > 1.20 and <0.8333, 268 differentially expressed proteins (DEPs) were screened; the numbers of upregulated and downregulated proteins were 178 and 90, respectively (Figure 4b). After the functional description of differential proteins by the Uniprot database, they were classified into six categories: metabolism (46.15%), cytoskeleton (11.24%), transport (10.06%), gene expression (9.47%), immune and oxidative stress (9.47%) and others (13.61%) (Figure 4c). 

**Figure 5:**
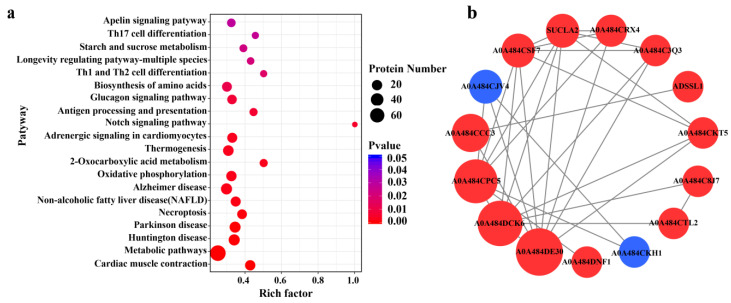
Pathway statistics for significant enrichment of differential proteins (**a**) and differential protein interaction network (**b**). The enrichment factor of X-axis in figure a represents the number of differential proteins annotated to the pathway/all identified proteins annotated to the pathway. The larger the value, the greater the proportion of differential proteins annotated to the pathway. The size of the dots in figure (**a**) indicates the number of differential proteins annotated to the pathway, while the color indicates the degree of significant difference in differential proteins. Red in figure (**b**) indicates upregulated proteins, while blue denotes downregulated proteins. The size of the circles indicates the intensity of the relationship. A0A484DE30: pyruvate dehydrogenase E1component subunit beta; A0A484DCK6: ATP synthase subunit alpha; A0A484CPC5: phosphoglycerate kinase; A0A484CCC3: aspartate aminotransferase; A0A484CJV4: elongation factor 1-alpha; A0A484CSF7: NADH dehydrogenase; SUCLA2: succinate-CoA ligase; A0A484CRX4: pyruvate dehydrogenase E1component subunit alpha; A0A484C3Q3: aconitate hydratase; ADSSL1: adenylosuccinate synthetase; A0A484CKT5: aconitate hydratase; A0A484C8J7: alpha-1, 4 glucan phosphorylase; A0A484CTL2: alpha-1, 4 glucan phosphorylase; A0A484DNF1: protein disulfide-isomerase; A0A484CKH1: 60S ribosomal protein L30.

A total of 236 pathways were identified, and the top 20 were selected to plot the statistics of pathways significantly enriched by DEPs (Figure 5a). We found that the metabolic pathways were the most enriched. To understand the interactions between DEPs, the DEPs were analyzed using the String database, and the DEPs interaction network map was plotted using the Cytoscape software (version 3.9.1) (Figure 5b). We found that among the interacting proteins, pyruvate dehydrogenase has the most dense relationship with other proteins.

**Figure 6:**
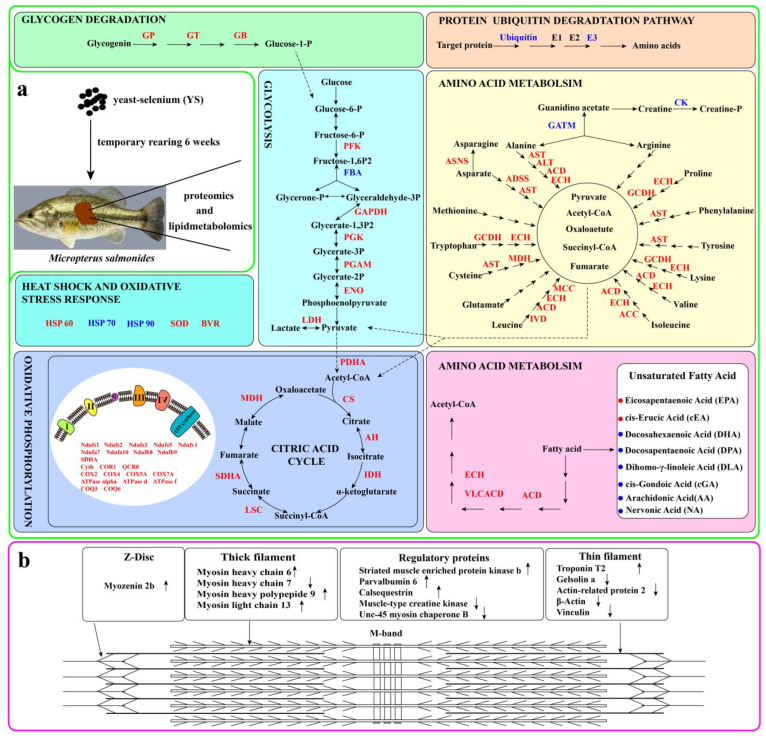
Schematic diagram of the effects of YS on enzymes related to protein, lipid and saccharide metabolism in *M. salmoides* (**a**) and schematic diagram of the primary components of striated muscle sarcomere (**b**). In (**a**), red indicates upregulated proteins, while blue indicates downregulated proteins. Red dots represent upregulated unsaturated fatty acids while blue dots denote downregulated unsaturated fatty acids. In (**b**), upward arrows indicate upregulated protein expression, while downward arrows indicate downregulated protein expression. GP: alpha-1, 4 glucan phosphorylase; GT: glucanotransferase; GB: glucan branching enzyme; PFK: 6-phosphofructokinase; FBA: fructose-bisphosphate aldolase; GAPDH: glyceraldehyde 3-phosphate dehydrogenase; PGK: phosphoglycerate kinase; ENO: enolase; LDH: L-lactate dehydrogenase; ACD: acyl-CoA dehydrogenase; VLCAD: very long chain acyl-CoA dehydrogenase; ECH: enoyl-CoA hydratase; PDHA: pyruvate dehydrogenase E1 component; CS: citrate synthase; ACO: aconitate hydratase; IDH: isocitrate dehydrogenase; LSC: succinyl-CoA synthetase alpha subunit; SDHA: succinate dehydrogenase (ubiquinone) flavoprotein subunit; MDH: malate dehydrogenase; NADH-quinone oxidoreductase subunit a; AST: aspartate aminotransferase; ALT: alanine transaminase; ACD: acyl-CoA dehydrogenase; ECH: enoyl-CoA hydratase; ASNA: asparagine synthase; ADSS: adenylosuccinate synthase; GCDH: glutaryl-CoA dehydrogenase; MCC: 3-methylcrotonyl-CoA carboxylase alpha subunit; GATM: glycine amidinotransferase; CK: creatine kinase; E2: ubiquitin-conjugating enzyme; E3: UBA domain containing 1; Ndufs1, Ndufs2, Ndufs3, Ndufs5, Ndufv1, Ndufa7, Ndufa10, Ndufb8 and Ndufb9 denote the component subunit of complex enzyme I; SDHA represents complex enzyme II; Cyt b, COR1 and QCR3 are the component subunit of complex enzyme III; COX2, COX4 and COX5A denote the component subunit of complex enzyme IV; ATPase alpha, ATPase d and ATPase f are the component subunit of ATPase. COQ3: ubiquinone biosynthesis O-methyltransferase; COQ6: ubiquinone biosynthesis monooxygenase.

Figure 6a presents the effects of YS addition on the metabolism of carbohydrates, lipids and amino acids as well as on the oxidative stress response in *M. salmoides*. Specifically, the expression of α-1, 4 glucan phosphorylase, 4-α-glucanotransferase and α-1, 4 glucan branching enzyme, which are the key enzymes in the glycogen degradation pathway, were upregulated. In contrast, 6-phosphofructokinase in the glycolytic pathway and citrate synthase, isocitrate dehydrogenase and succinate-CoA ligase in the tricarboxylic acid (TCA) pathway were upregulated. Figure 6b shows the differential proteins of the striated muscle sarcomere, including myosin heavy chain 6, myosin heavy chain 7, myosin heavy chain 9, myosin light chain 13 and myozenin 2b.

## 3. Discussion

### 3.1. Effects of YS on Muscle Carbohydrate and Lipid Metabolism

The analysis of the experimental results revealed that the expression of three key enzymes (α-1, 4 glucan phosphorylase, 4-α-glucanotransferase and α-1, 4 glucan branching enzyme) associated with glycogen degradation and six enzymes (6-phosphofructokinase, glyceraldehyde-3-phosphate dehydrogenase, phosphoglycerate kinase, phosphoglycerate mutase, enolase and L-lactate dehydrogenase) involved in the glycolytic process were upregulated in the fish from the YS group. It has been reported that fasting decreases the expression of genes related to the glycolytic pathway as well as the activity of key enzymes in fish [17]. However, the present study showed that YS addition enhanced the glycolytic pathway in *M. salmoides*. This was consistent with the findings of Naderi et al. on rainbow trout [10]. Furthermore, fructose-bisphosphate aldolase expression was downregulated in the fish from the YS group, suggesting that YS addition may lead to the accumulation of fructose 1, 6-diphosphate in the glycolytic pathway in *M. salmoides*. Meanwhile, our findings showed that the expression of pyruvate dehydrogenase E1 component subunit α was upregulated. This enzyme is a component of the pyruvate dehydrogenase complex, which is essential for the entry of sugar, lipid and amino acid metabolites into the TCA cycle [18]. The expression of citrate synthase, aconitate hydratase, isocitrate dehydrogenase, succinate dehydrogenase, succinate-CoA ligase and malic enzyme were upregulated in the TCA cycle, indicating that YS addition promoted the catabolism of glycogen and lipids to meet the energy requirements of the fish under temporary rearing.

Fasting triggers the mobilization of the body stores of fish such as glycogen, lipids and proteins to generate energy [19]. In the present study, the expression of acyl-CoA dehydrogenase, hydroxyacyl-CoA dehydrogenase and acetyl-CoA acyltransferase were upregulated, and that of 3-hydroxyacyl-CoA dehydratase was downregulated in the fish from the YS group. These are key enzymes in the fatty acid β-oxidation cycle and fatty acid synthesis pathway [20]. This finding suggested that YS addition enhanced fatty acid catabolism and attenuated fatty acid synthesis in *M. salmoides*, ultimately leading to a reduction in the fat content. This was consistent with the report of a study that demonstrated that the supplementation of Se nanoparticles enhanced the expression of genes related to fatty acid β-oxidation in grass carp [21]. Furthermore, the levels of EPA and CEA increased in the fish from the YS group, while those of DPA, DHA, NA, DGLA, and CGA decreased, indicating that YS addition also altered the unsaturated fatty acid metabolic profile of *M. salmoides*.

### 3.2. Effects of YS on Muscle Protein and Amino Acid Metabolism

Protein degradation is divided into enzymatic (ubiquitin-proteasome) and non-enzymatic (lysosome) pathways, and under starvation conditions, muscle protein degradation relies more on the ubiquitin-proteasome pathway, which is also the main pathway associated with muscle atrophy [22]. In the present study, the expression of fish ubiquitin and ubiquitin-protein ligases was downregulated in the YS group, indicating that YS addition during fasting attenuated protein degradation. This was consistent with the study of Wang et al., which demonstrated that dietary YS inhibits the intracellular ubiquitin proteasome pathway, thereby inhibiting protein degradation in the muscle [23]. Furthermore, the expression of S-adenosylmethionine synthase (SAMS) was downregulated in the fish from the YS group. SAMS is an enzyme that catalyzes the formation of S-adenosylmethionine, an active form of methionine, from methionine and ATP. Methionine is susceptible to free radical attack, which alters its protein structure [24]. Meanwhile, the expression of protein disulfide isomerase and peptidyl-prolyl cis-trans isomerase in the fish from the YS group was upregulated. They prevent protein misfolding and promote correct folding [25]. The aforementioned findings suggested that YS addition mitigated protein damage induced by starvation and thus helped to improve the stress capacity of bass.

### 3.3. Effects of YS on Muscle Energy Metabolism

NADH ubiquinone oxidoreductase, cytochrome c oxidase, electron transfer flavoprotein dehydrogenase and cytochrome b are involved in the transfer of electrons in the respiratory chain in the mitochondria. ATP synthase subunit d is a constituent subunit of ATP synthase; the latter uses free energy stored in the transmembrane ion gradient to generate ATP [26]. Solute carrier family 25 is a family of proteins of mitochondrial carriers that transport metabolites, nucleotides and cofactors across the inner mitochondrial membrane, connecting the cytosol and mitochondrial matrix [27]. In this study, we found that the expression of all these enzymes was increased in the YS group compared with that in the control group. This indicated that YS addition under temporary cultivation enhanced the oxidative phosphorylation pathway in *M. salmoides*, supporting the fact that saccharide and lipid catabolism were enhanced to meet the energy requirements of the body. However, it has been reported that animals under starvation reduce their metabolic demand for a greater chance of survival and that a temporarily reduced mitochondrial energy demand may lead to an increase in reactive oxygen levels [28]. The enhancement of oxidative phosphorylation processes due to the addition of Se may be an adaptive and protective mechanism of *M. salmoides*. Furthermore, ubiquinone biosynthesis O-methyltransferase and ubiquinone biosynthesis monooxygenase are involved in the O-methylation and monooxygenation steps in ubiquinone biosynthesis, respectively [29]. The present study indicated that the expression of these two enzymes was upregulated in the fish from the YS group, suggesting that the addition of Se may lead to an increase in ubiquinone to meet electron transfer during enhanced energy metabolism. Ubiquinone can also scavenge free radicals and protect cells from oxidative damage [30], and this improves the antioxidant capacity of *M. salmoides*.

### 3.4. Effects of YS on Muscle Tissue Structure

Muscle hardness, chewiness and elasticity are important parameters for evaluating fish quality. These factors are closely related to the structural characteristics of muscle fibers, and they are negatively correlated with muscle fiber diameter and positively correlated with muscle fiber density [31]. It has been indicated that the muscles of wild fish usually have higher hardness and elasticity values compared with farmed fish, which are more attractive to consumers [32]. Our previous study indicated that YS addition under fasting conditions with temporary cultivation improved muscle hardness, chewiness and elasticity in *M. salmoides* [6]. The muscle fiber microstructure of the bass from the YS group in this study was more tightly arranged and smaller in diameter, further demonstrating that YS had a positive effect on improving the quality of *M. salmonides*.

Myofibrillar proteins largely determine the structural characteristics of muscles. They are primarily composed of thick filaments (myosin), thin filaments (actin, tropomyosin and troponin), Z-discs and M-wires [33]. This study identified some proteins related to the aforementioned proteins. The addition of YS resulted in the upregulation of MHC 6, MHC 9 and MHC 13 expression and the downregulation of gelsolin a, Unc-45 myosin chaperone b, actin-related protein 2 and beta actin expression in the fish, indicating that the addition of Se affected the myofibril structure. Specifically, gelsolin a and actin-related protein 2, which play important roles in actin assembly, are involved in actin remodeling and dynamic changes in the cytoskeleton [34]. Under a state where fish growth is inhibited, the disruption of actin may have adverse effects on fish muscle structure. It is hypothesized that YS attenuates the depolymerization of actin in fish caused by fasting stress, thereby stabilizing the actin network and protecting the muscle tissue structure.

Furthermore, human and mouse studies have suggested that the mutations of regulatory proteins associated with myocyte activity, such as myozenin 2, could cause myofibril structural disorders and an abnormal Z-disk structure, resulting in myocyte injury [35]. An increased level of muscle-type creatine kinase is a marker of muscle injury under stressful conditions, such as fasting [36]. In the present study, YS addition resulted in an upregulation of the expression of myozenin 2 and a downregulation of creatine kinase, suggesting that YS addition may alleviate the damage caused by fasting stress in *M. salmoides* muscles. Striated-muscle-enriched protein kinase b is involved in muscle cell signaling and is closely related to muscle mass. It plays a regulatory role in protein synthesis and an inhibitory role in protein hydrolysis. Its activity is controlled by the reversible phosphorylation of its serine and threonine residues [37]. In the present study, the expression of striated-muscle-enriched protein kinase b, serine/threonine protein phosphatase and non-specific serine/threonine protein kinase in the fish from the YS group was upregulated, indicating that YS could promote protein synthesis and improve muscle quality. The concentration of parvalbumin showed a positive correlation with the absolute rates of contraction and relaxation of skeletal muscle. Moreover, the expression of parvalbumin was upregulated in the fish from the YS group, which was contrary to the findings that the expression of parvalbumin in fine-scale butterfish was decreased under fasting conditions [38]. We hypothesized that YS addition could attenuate the negative effects of fasting on muscle contraction and diastole. In summary, YS addition had an ameliorative effect on muscle protein degradation and muscle damage in the fish caused by fasting stress.

### 3.5. Effects of YS on Muscle Immunity and Oxidative Stress Response

The quality of a fish is closely related to its antioxidant capacity, and its muscles are susceptible to oxidative stress, leading to degradation. In this study, we found that the expression of superoxide dismutase (SOD) and biliverdin reductase (BVR) were upregulated in the YS group, indicating that YS addition enhanced the oxidative stress capacity of the bass. SOD can effectively scavenge superoxide, while BVR can catalyze the conversion of biliverdin to bilirubin; they both play an important role in the antioxidant defense mechanism of the body [39]. The expression of NAD-dependent protein deacylase was upregulated in the YS group. This protein belongs to the sirtuin family and plays a key role in regulating the cellular antioxidant and redox signaling pathways [40]. It is hypothesized that YS may regulate the redox pathway in fish. In this study, compared with the control group, the expression of neurocalcin delta b was downregulated in the YS group, whereas that of neurofilament light polypeptide b (NEFL) and glutaryl-CoA dehydrogenase was increased (GCDH). Neurocalcin delta b is a neuronal calcium sensor, and its deficiency may lead to muscle atrophy or weakness [41]. NEFL and GCDH can alleviate muscle damage [42,43]; therefore, it is difficult to determine if Se is effective in alleviating muscle damage caused by nerve injury under stressful conditions. Furthermore, an increased synthesis of heat shock protein (HSP) is an important manifestation of stress response in fish [44]. However, in this study, the expression of HSP60 was upregulated in the YS group, whereas that of HSP70 and HSP90 were downregulated, suggesting that the expression of the HSP family members could differ even in the presence of a stress response.

## 4. Materials and Methods

### 4.1. Temporary Rearing of Micropterus Salmoides and Preparation of Muscle Samples

*M. salmoides* (*n* = 150) were purchased from Baishazhou Agricultural Products Market in Wuhan (Hubei, China) with an average initial weight of 650 ± 33.5 g/fish. YS (1 kg containing 2000 mg Se) was purchased from Angel Yeast Co., Ltd. (Yichang, China). The organic selenium (selenocysteine and selenomethionine) content of the YS was about 98%. A total of 150 *M. salmoides* were randomly and equally divided into blank and YS groups (treated with 1 mg/L YS in water containing Se 0.002 mg/L), and each group consisted of 3 tanks with 25 fish in each tank. They were subjected to 6 weeks of temporary fasting conditions in a 600 L recirculating tank. Before the experiment, 500 L of tap water was added to each recirculation tank, and aeration was performed for 24 h to remove Cl^−^ from the water and to ensure the adequacy of dissolved oxygen. Moreover, 1 mL/L of 5% povidone iodine was added as a disinfectant. During the experiment, the tap water was changed every week. Accordingly, the same concentration of YS was added to the YS groups. Furthermore, day and night scenarios were simulated, the water temperature was maintained at 16–18 °C and the dissolved oxygen level in the water was >5.0 mg/L. In addition, the concentration of ammonia nitrogen in the water was approximately 0.02 mg/L.

At the sixth week of temporary rearing, eight fish samples were randomly selected from each tank and evenly divided into two parts for lipid metabolomics and proteomics analysis. All fish were anesthetized with tricaine methanesulfonate (250 mg/L) and then placed on ice to collect dorsal muscles as test samples. The obtained muscle samples were snap-frozen in liquid nitrogen and stored at −80 °C for subsequent analysis.

### 4.2. Observation of Muscle Tissues

Dorsal muscles were collected and fixed in 10% formalin, dehydrated in ethanol, equilibrated in xylene and then embedded in paraffin. Then, the samples were sectioned for a thickness of approximately 8 μm and stained with hematoxylin and eosin. The muscle fiber microstructure was observed by using Pannoramic 250 Flash microscope slide scanner (3DHIESTECH, Budapest, Hungary), a Regulus 8100 scanning electron microscope (Hitachi, Tokyo, Japan) and an H-700 transmission electron microscope (Hitachi, Tokyo, Japan).

### 4.3. Non-Targeted Lipid Metabolomics Analysis Based on Liquid Chromatography-Mass Spectrometry

#### 4.3.1. Preparation of Lipid Samples

Lipid samples were extracted according to a previous method [45], albeit with minor modifications. Briefly, 25 mg of muscle tissue was weighed in a centrifuge tube; 800 μL of cold dichloromethane/methanol (3:1) was added, ground for 5 min and frozen overnight. Next, the supernatant was removed by centrifugation at 25,000× *g* and 4 °C for 15 min (repeated once). The supernatant was lyophilized and re-dissolved in isopropanol, acetonitrile and water (2:1:1), which was followed by centrifugation for 20 min. The supernatant was then collected and analyzed using a 2777C UPLC liquid chromatograph system (Waters Corporation, Manchester, UK) and a Xevo G2-XS QTOF mass spectrometer (Waters Corporation, Manchester, UK). Furthermore, equal amounts of quality control samples were prepared to evaluate the stability of the instrument analysis.

#### 4.3.2. Mass Spectrometric Detection

Lipomics detection was supported by a Huada BGI device (Wuhan, Hubei, China). A quality control (QC) sample consisting of a mixture of equal aliquots of all samples included in this study was injected into every ten samples. After separation using a chromatographic column, the samples were directly introduced into the mass spectrometer for detection. An ACQUITY UPLC CSH C18 column (100 mm × 2.1 mm, 1.7 μm, Waters, Manchester, UK) was used for the chromatographic separation. The column temperature was 55 °C, and the flow rate was 0.4 mL/min. The A mobile phase was ACN: H_2_O = 60:40, and the B mobile phase was IPA: ACN = 90:10, both containing 0.1% FA and 10 mM ammonia formate. The metabolites were eluted using the following gradients: 0–2 min, 40–43% mobile phase B; 2.1–7 min, 50–54% mobile phase B; 7.1–13 min, 70–90% mobile phase B; and 13.1–15 min, 40% mobile phase B. The injection volume was 5 μL. Mass spectrometry was performed in the positive ion (ESI^+^) and negative ion (ESI^−^) modes. The capillary and cone voltages in the ESI^+^ mode were 3.0 kV and 40.0 V, respectively, while the capillary and cone voltages in the ESI^−^ mode were 2.0 kV and 40.0 V, respectively. The MSE mode was used for centroid data acquisition with a scanning range of 100–2000 Da for positive ions at the first level and 50–2000 Da for negative ions and with a scanning duration of 0.2 s. All parent ions were fragmented at energies from 19 to 45 eV, and all fragmentation information was collected with a scanning time of 0.2 s. During data acquisition, real-time quality correction was performed every 3 s for the LE signal.

#### 4.3.3. Data Processing

Peak extraction, alignment, normalization and compound identification were performed with the Progenesis QI (version 2.2) software. Data preprocessing was performed using the metaX software, including filling in missing values and removing low-mass ions (removing more than 50% of the missing ions in the QC samples or more than 80% of the missing ions in the actual samples), and then ions with a relative standard deviation greater than 30% in the QC samples were filtered out. Partial least squares discriminant analysis (PLS-DA) was performed using the SIMCA software (version 13.0) to derive the variable importance for the projection (VIP). Differential ions were screened based on VIP > 1, difference multiples > 1.2 or <0.8333 and *p*-value < 0.05 (Student’s *t*-test). The difference ions were annotated using the Lipid Maps database. Lipids were annotated with a maximum tolerance of 10 ppm.

### 4.4. Isobaric Tags for Relative- and Absolute-Quantitation-Based Proteomics Analysis

#### 4.4.1. Extraction and Detection of Protein Samples

Muscle proteomics analysis was performed according to a method described in previous studies [46], albeit with minor modifications. Dorsal muscle samples were used for protein extraction, and the Bradford method was used to measure the total concentration of protein extracts. Each sample protein was digested with trypsin at a ratio of 40:1 for 4 h at 37 °C. Next, the enzyme was added once the sample reached the same ratio, and the digestion was continued for 8 h. The digested products were desalted using a Strata X column and dried under vacuum. Quantitative proteomic analysis was performed using isobaric tags for relative and absolute quantitation (iTRAQ) technology. The polypeptides in the six samples (control and YS groups, each containing three biological replicates) were labeled with iTRAQ 8-plex reagent. The peptides were separated using a Shimadzu HPLC Pump System (LC-20AB, Shimadzu, Kyoto, Japan) and a Nano-HPLC instrument (Thermo Fisher Scientific, San Jose, CA, USA), which was followed by detection with a Q-Exactive HF-X tandem mass spectrometer (Thermo Fisher Scientific, San Jose, CA, USA).

#### 4.4.2. Data Processing

The Mascot software (version 2.3.02) was used to compare the corresponding databases for protein identification. Protein quantitative analysis was performed using the IQuant software. To assess the credibility of the peptide, filtration with 1% FDR was performed at the peptide-spectrum matches (PSM) level. The identified peptide sequences were then assembled into a set of trusted proteins based on the parsimony principle. In order to control the false positive rate of the protein, filtration with 1% FDR was performed at the protein level. Differential proteins were screened for difference multiples > 1.20 or <0.8333, and a *p*-value < 0.05 (Student’s *t*-test) indicated a significant difference. Proteins were annotated using the Gene Ontology (GO, http://geneontology.org/) (accessed on 10 January 2023), Uniprot (https://www.uniprot.org/) (accessed on 15 January 2023) and KEGG (http://www.genome.jp/kegg/) (accessed on 2 February 2023) databases.

## 5. Conclusions

In this study, it was found that the treatment of *M. salmoides* with YS (Se 0.002 mg/L) under temporary fasting conditions for 6 weeks resulted in a tighter arrangement of fish muscle fibers and an increased flesh hardness. The results indicated that YS addition enhanced the muscle glycogen degradation, glycolysis and fatty acid oxidation pathways. Meanwhile, the expression of key enzymes in the TCA cycle and the oxidative phosphorylation pathway was increased in the fish flesh from the YS group in order to meet the energy requirements of the *M. salmoides* under the fasting state. The downregulated expression of ubiquitin and ubiquitin ligase in the fish flesh from the YS group mitigated protein degradation. At the level of muscle structural proteins, YS had an ameliorating effect on fasting-induced muscle protein degradation and damage. Furthermore, YS enhanced the resistance to oxidative stress in the *M. salmoides*. Organoselenium can improve the muscle quality of bass through the metabolic, protein, immune and antioxidant pathways.

## Data Availability

Data are available upon request.
